# Nanofabrication and coloration study of artificial *Morpho* butterfly wings with aligned lamellae layers

**DOI:** 10.1038/srep16637

**Published:** 2015-11-18

**Authors:** Sichao Zhang, Yifang Chen

**Affiliations:** 1 Nanolithography and Application Research Group, School of Information Science and Engineering, Fudan University, Shanghai 200433, China

## Abstract

The bright and iridescent blue color from *Morpho* butterfly wings has attracted worldwide attentions to explore its mysterious nature for long time. Although the physics of structural color by the nanophotonic structures built on the wing scales has been well established, replications of the wing structure by standard top-down lithography still remains a challenge. This paper reports a technical breakthrough to mimic the blue color of *Morpho* butterfly wings, by developing a novel nanofabrication process, based on electron beam lithography combined with alternate PMMA/LOR development/dissolution, for photonic structures with aligned lamellae multilayers in colorless polymers. The relationship between the coloration and geometric dimensions as well as shapes is systematically analyzed by solving Maxwell’s Equations with a finite domain time difference simulator. Careful characterization of the mimicked blue by spectral measurements under both normal and oblique angles are carried out. Structural color in blue reflected by the fabricated wing scales, is demonstrated and further extended to green as an application exercise of the new technique. The effects of the regularity in the replicas on coloration are analyzed. In principle, this approach establishes a starting point for mimicking structural colors beyond the blue in *Morpho* butterfly wings.

Structural color is frequently seen in butterflies^1^,^2^,^3^,^4^,^5^, beetles^6^, and sea animals^7^,^8^, etc. Among them, the most widely cited examples are the *Morpho* butterflies^9^,^10^,^11^,^12^, living in South America. The coloration of the butterfly wings exhibits a number unique features such as broad blue iridescence, brilliant luster, speckle-like aspects, high resistance to discoloration, high sensitivity to environment and angle independent spectra^13^,^14^. Since the first observation of the inside structure with powerful scanning electron microscope (SEM)^15^, substantial researches on the origin of the coloration by the elaborate nanostructures in *Morpho* butterfly wings have been widely conducted^16^,^17^,^18^, driven by extensively potential applications. The anticipated applications include iridescent textile apparel^19^, functional coatings^20^, unmatchable color security coding^21^, efficient solar cells^22^, highly selective gas sensors^23^,^24^, chemical sensors with excellent sensitivity and selectivity^25^,^26^ and high-speed infrared imaging devices^27^,^28^, etc. The key to the success in interpreting the coloration precisely is to establish a reliable technical methodology for the architecture of the periodical structure on the micro- and nanoscale on butterfly wing scale, which is also an inevitable step toward its applications in daily life. As schematically shown by the original architecture in Fig. 1a, the wing scale is covered with parallel ridges with random heights from each other. Presenting on the sidewall of each ridge is actually lamellar structure consisting of alternate layers of cuticle and air. Such kind of nanostructures has been a daunting challenge in replication because of the 3D variations in the profile. Even though, numerous attempts have been reported. Saito *et al*.^29^,^30^ and Chung *et al*.^31^ mimicked the blue color with wide angular viewing by multilayer deposition of TiO_2_/SiO_2_ on irregular substructure. Watanabe *et al*.^32^ fabricated replica of *Morpho* butterfly scales and observed blue color reflection by using focused-ion-beam chemical-vapor-deposition (FIB-CVD). Huang *et al*.^33^, Zhang *et al*.^34^,^35^,^36^, Kang *et al*.^37^ and Chen *et al*.^38^,^39^ used butterfly as bio-templates to synthesize the 3D nanostructures by metal oxides or polydimethylsiloxane (PDMS) and reported that the replica can reflect different colors with various lattice sizes and refractive indices. However, all the reported approaches were not based on standard top-down nanolithography process with large area, high yield and low cost. Only one exception is that Aryal *et al*.^40^ recently introduces a method for the large area nanofabrication by industrialized techniques and subsequent nanoimprint of 3D butterfly wing scales. Unfortunately its optical coloration is not characterized. Moreover, most of the mimicked nanostructures uses inorganic materials, resulting in replicas’ displaying color unlike the real *Morpho* butterfly because optical properties of inorganic materials are different from that of cuticle in the real butterfly wing scales.

This paper reports a technical breakthrough, recently achieved in the fabrication of aligned lamellae multilayer on nanopillars, as schematically presented in Fig. 1b, to mimic the coloration of *Morpho* butterfly wing scales by a novel process based on electron beam lithography and alternate development/dissolution on PMMA/LOR superlattice multilayers. The whole artificial scale is covered by a periodical ridge grating with PMMA/Air branches. Basic coloration property of the artificial lamellae structures in both blue and green are demonstrated and their differences from real wings have been analyzed by solving Maxwell equations using a FDTD simulator. Angle-resolved spectra show abnormal red shifts instead of blue shift as in real wings, which is explained by the regularity of the ridge gratings in our case. The technical methodology developed in this work provides us with a great power to adjust branch width, layer number, layer thickness for desired colors, and even materials of the scales in the manufacturing of more colorful wings beyond the blue in *Morpho* butterfly.

## Results

### The coloration of the designed Simple configuration by numerical simulation

**Figure 1:**
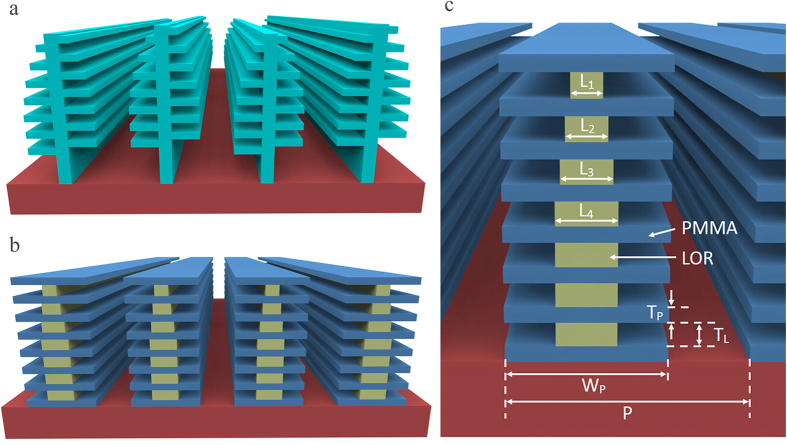
Schematic diagram for the *Morpho* butterfly wing scales. (**a**) The Original configuration similar to real wing scales with Christmas-tree shape and off-set lamellae layers. (**b**) The designed scales to be fabricated with aligned lamellae structures of PMMA/LOR alternate layers. (**c**) Definitions of dimension symbols used in the text.

The wing scale being constructed in this work is schematically shown in Fig. 1b. It is formed by a free standing periodical ridge grating with a fixed pitch of 1.3–1.4 μm. On each ridge as a single tree, there are aligned lamellae layers as alternate solid cuticle and cuticle-pillar-supported air layers to mimic *Morpho* butterfly wing scales. The geometry dimensions in 5 different lamellae configurations are summarized in Table 1. In the table, all the parameters are defined in Fig. 1c. The width of tree-branch is fixed as 850 nm for all the layers, leaving a 450-nm wide gap between two adjacent ridges. The reasonably wide gaps are designed to make sure the multilayer reflection happens within one ridge only, which is termed as quasi-multilayer reflection.

In this work, PMMA resist is used as the branch material and the ridge axis (pillar) is constituted by a PMMA/LOR multilayer, where LOR stands for lift-of-resist supplied by MicroChem Corp. The refractive indices of the PMMA (n_PMMA_ = 1.50) and LOR (n_LOR_ = 1.58) layers were measured by an interferometer. Both of the materials show no adsorption in visible light and are totally colorless. The thicknesses (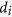) of the PMMA and LOR were calculated for a quarter-wave stack with a stop-band centre wavelength in blue (480 nm) and green (520 nm) spectral range, based on the constructive interference condition: 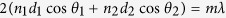
14, in which the footnote “1” and “2” denote PMMA and LOR, respectively, and 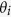 is the incident angle. For an ideal multilayer system with a reflective constructive interference at the wavelength of 480 nm, the thicknesses of the PMMA and air should be 80 nm and 120 nm, respectively, meaning that the thickness of the LOR is also 120 nm. In the same way, the thicknesses of the PMMA layer and LOR layer for green color should be 87 nm and 130 nm, respectively. Two different total-layer-numbers (counting both PMMA and LOR), 11 layers (5 periods) and 15 layers (7 periods) were designed and fabricated to experimentally reflect the effect of layer number on the reflectivity.

Coloration characters of the designed wing scale are first theoretically studied by modelling the reflectance spectra under both vertical and oblique incidence, utilizing finite-difference time domain (FDTD) method. Specific concerns are: the effects of the substrate material, the pillar shape, the layer number, the incidence/viewing angles and the regularity of the ridge gratings on the optical property.

**Figure 2:**
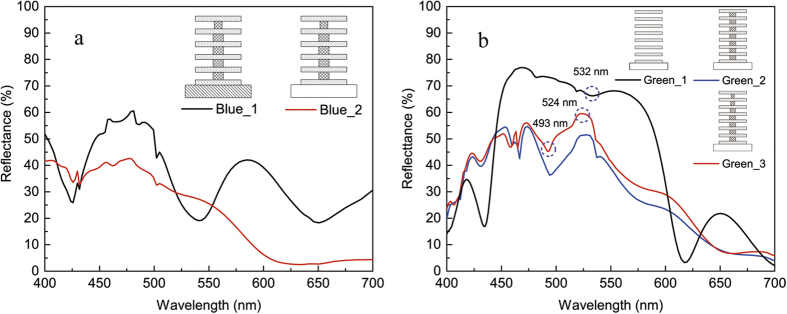
The FDTD simulation results of the spectra for the designed lamellae structures. (**a**) Two multilayer samples with totally 11 layers on silicon (Blue_1) and quartz (Blue_2), respectively. The strong peak around 580 nm in the spectrum from Blue_1 with silicon substrate shows clear substrate effect. (**b**) Three multilayer samples (Green_1, Green_2 and Green_3) with totally 15 layers on quartz. Note that the major difference of the three structures is the pillar shape. The electric field distributions on the three marked points were simulated. All the physical dimensions are listed in Table 1. For detailed explanations, see the text.

Figure 2 presents the simulated reflectance of the designed lamellae layers. Figure 2a shows the results from the samples with 11 multilayers on Si (Blue 1) and quartz (Blue 2), respectively. Both of them show a strong reflection peak around 480 nm (blue color) as desired. But, the spectrum with the silicon as the substrate shows an extra peak around 590 nm (yellow color), which is caused by the specular reflection in the PMMA/Si interface. This reflection is greatly reduced in PMMA/quartz interface because of the small difference in refractive index between PMMA and quartz, resulting in the blue color only.

**Figure 3:**
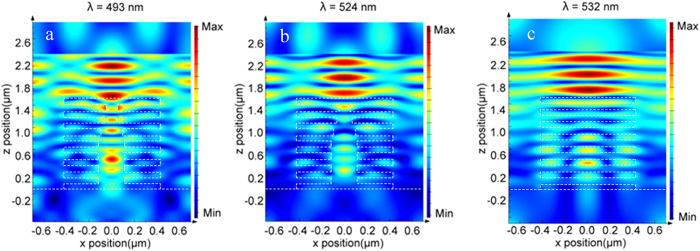
The FDTD simulations of spatial distributions of the electric field, E2 for the three wavelengths in Figure 3b. (**a**,**b**) correspond to the wavelengths at 493 and 524 nm, respectively, in Green_3. The strongest travelling mode seen in the PMMA/LOR pillar in (**a**) is responsible for the reflection dip at 493 nm in the spectra (both the red and the blue line) in figure 3b. The relatively weak E^2^ in the multilayer in (**b**) (524 nm) and (**c**) (532 nm) explains the high reflection in the spectra. The dash lines highlight the lamellae structures.

The effect of the pillar shape on the coloration is also studied as demonstrated in Fig. 2b. Three different shapes of the pillar have been compared as shown in the inset of Fig. 2b, corresponding to (1) no pillar, (2) 230-nm wide pillar, (3) trapezium shape on the first three layers from the top and 230-nm wide for the rest, which is similar to the fabricated ridges. The spectrum without the pillar (the black line) is typically the reflectance of a 1D photonic crystal with high reflectivity in 450 nm–600 nm. Addition of 230-nm wide pillars (the blue line) or sharp pillars (the red line) do modify the reflectance, but no big difference in the reflectance is made by the two pillar shapes. Both of them is in the same color band with only a slight change in the reflectivity. The big dips around 493 nm are caused by resonant transmission modes in the PMMA/LOR 1D crystal. The simulated electric field distribution, E^2^ also proves the existence of propagating modes in the pillar as shown in Fig. 3a, leading to the reduction in reflectance. The E^2^ distributions corresponding to the two wavelengths at 524 nm (Fig. 3b) and 532 nm (Fig. 3c), respectively, show relatively weak transmission through the pillars than the one at 493 nm, corresponding to reflection peaks in the spectra.

### The fabricated lamellae structures by a novel process

**Figure 4:**
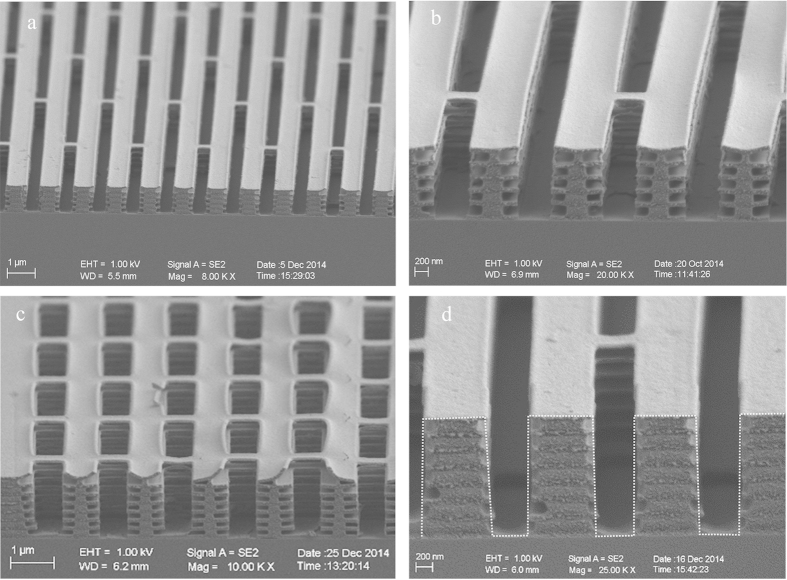
The micrographs of scanning electron microscope (SEM) for fabricated wing scales with aligned lamellae multilayers. (**a**) An overview of the mimicked scale with low magnification. (**b**) A close-up view of the cross-section of the 11-layer lamellae structure. (**c**) The cross-sectional view of the 15 layers structure. The top PMMA layer was bent up in the cleaving of the sample. (**d**) The ridge grating as highlighted by dash-lines.

A novel process by electron beam lithography with alternate development/dissolution on PMMA/LOR superlattice multilayers has been developed for the fabrication of aligned lamellae structures. The most important procedure of the process is to create the air-layers by a diluted alkali solution through selective dissolution on the LOR layer with controllable manner^41^. Detailed description of the processing study is shown in the Method section. Figure 4 presents SEM micrographs of the fabricated aligned lamella structures for blue color with 11 layers and green color with 15 layers, respectively, on Si substrate. All the resultant dimensions are very close to designed figures as listed in Table 1. The PMMA/LOR pillars are broad at the bottom and narrow on the top, similar to that in real *Morpho* butterfly wing scale. To distinguish the color by the quasi-multilayer reflection through the lamellae layers from that through the ridge gratings (as highlighted by the dash-lines in Fig. 4d), lamella structures with the same pitch and the same height but short undercuts were also fabricated.

### Spectral characterizations of colorations

**Figure 5:**
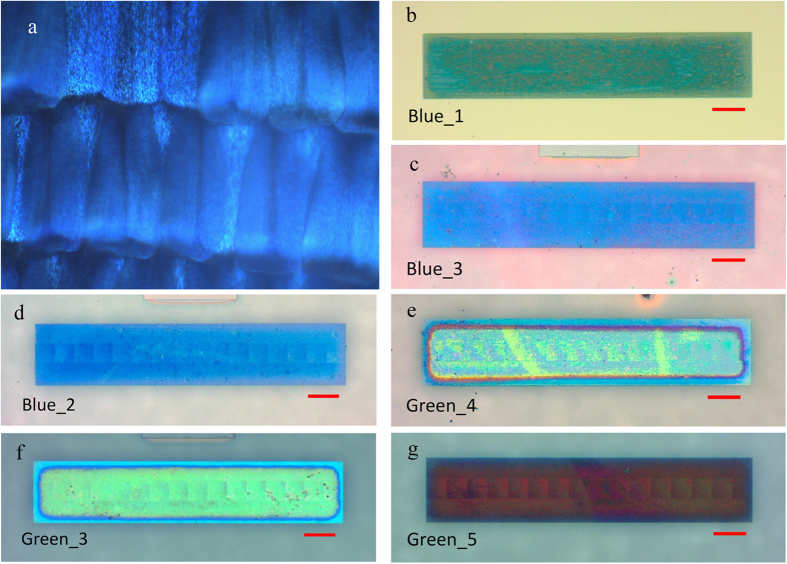
Optical microscope images for structural colors from patterned areas as artificial wing scales. (**a**) The image from the scale of a real *Morpho didius* butterfly wing. (**b**) The color image from an 11-layer lamellae structure on Si, showing the complementary color of blue and green. (**c**) The image from the same pattern as in (**b**) but on quartz substrate with slightly thinner layer thicknesses than designed values, showing purer blue. (**d**) The image of patterned area with the same layer structure as above and corrected thicknesses, showing the blue closer to the real one in (**a**). (**e**,**f**) The images from patterned areas for green color with 11 layers and 15 layers, respectively. (**g**) The image showing dark red color from the patterned area with 1.5-μm pitched ridge gratings without PMMA/Air branches. The scaling bar in all the images is 100 μm.

**Figure 6:**
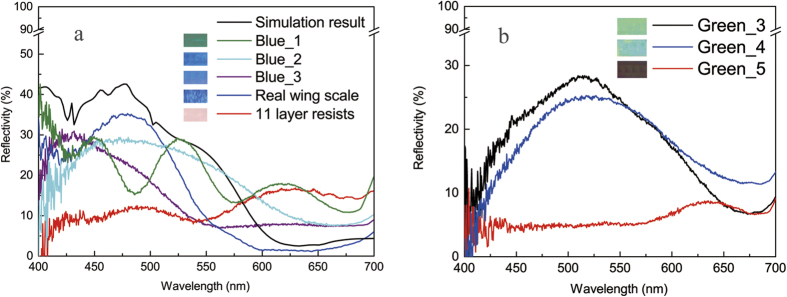
The measured and the simulated reflectance spectra. (**a**) The reflectivity from the naturel, the fabricated and the simulated wing scales with blue color. The displayed colors in the inset are from microscope images. (**b**) The reflectivity from the fabricated scales with green color. The sample names and their corresponding colors are shown in the inset. The red line with low reflectivity was taken from a ridge grating without PMMA/Air branches. Its color is dark red at 620 nm. For detailed explanations, see the text.

The structural color from fabricated artificial wing scales were characterized by spectral method in visible region with both vertical and oblique incidence of light. Figure 5 presents a series of optical microscope images showing the colors from the patterned areas. Also presented in the figure is the image from the wing scale of a real *Morpho didius* butterfly for comparison (Fig. 5a). Figure 5b shows the color from an initially fabricated sample (Blue_1) with 5 periods (totally 11 layers) on Si substrate. Both aquamarine and green color can be vaguely observed. A vertical reflectance spectrum (the green line in Fig. 6a) in visible region (400–700 nm) was scanned on Blue_1 by a NOVA-EX spectrometer (Ideaoptics Instruments Co. Ltd., China) under a collimated source emitting white plane wave. It can be seen that the spectrum has components in both blue region (450 nm) and green region (530 nm). The blue peak is due to the quasi multilayer reflection in PMMA/Air branches. The green peak is the contribution from the PMMA/Si interface by specular reflection. When a quartz was used as the substrate in sample Blue_3, much purer blue color without the red component is observed from the image in Fig. 5c. The corresponding spectrum (purple line in Fig. 6a) also shows the disappearance of the green peak. Therefore, the substrate material, characterized by the refractive index, is important to ensure the contribution from the substrate is limited. However, more careful inspection found that although the spectrum mainly cover the blue region of 400–540 nm, the maxima is actually around 430 nm instead of the desired 480 nm. This 50 nm blue shift might be caused by the deviations in resist thicknesses from the designed. Further fabrication of lamellae layers (Blue_2) by using corrected thicknesses of PMMA and LOR gives rise to correct reflectance peak at 480 nm as shown by the cyan line in Fig. 6a. This further proves that the observed blue color is truly through the reflection in the artificial lamella multilayers.

Also included in Fig. 6a is the spectrum taken from real *Morpho didius* butterfly wing (blue line), which agrees with both the simulated and measured reflectance using the designed structure as discussed above. This suggests the structural model established in this work mimics the major coloring characters of the butterfly wing scale very well. The spectrum appearing as the red line in Fig. 6a is taken from the multilayer of PMMA/LOR with totally 11 layers without patterning, showing very low reflectivity in the blue region, whose co7 pntribution can be ignored.

By the same way, green color is also mimicked by redesigning the layer thicknesses of both PMMA and LOR. The spectra measured from the fabricated samples with 5 periods (sample Green_3) and eriods (sample Green_4) respectively, are presented in Fig. 6b. Both reflection peaks are at the desired 520 nm for green color, which is also proved by the optical images of the patterned areas with lamellae structures in Fig. 5e,f, respectively. Both optical images and spectra demonstrate that the fabricated lamellae structures with 7 periods exhibits higher brightness and contrast than that with 5 periods. The spectrum taken from Green_5 as ridge gratings with negligible PMMA/Air branch-widths (Fig. 4d) shows no contribution to green color apart from a very weak peak at 630 nm, which is consistent to the optical image in Fig. 5g.

### Angle-resolved spectra

**Figure 7:**
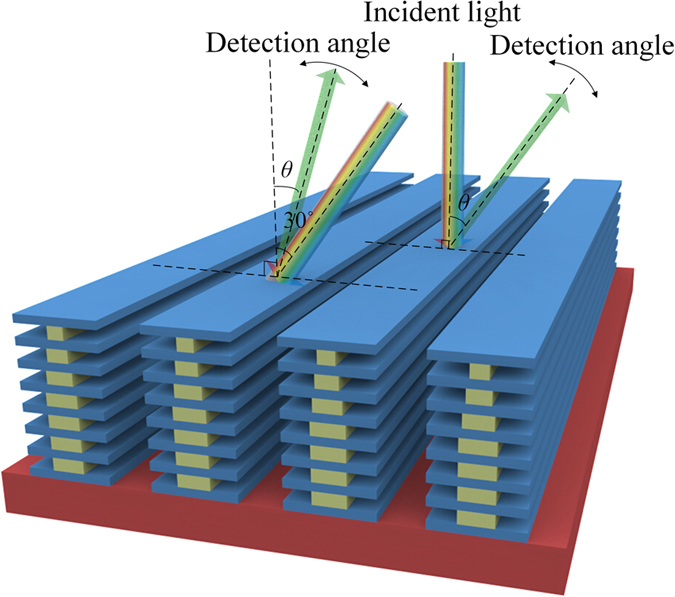
The schematic diagram for the light illumination with normal incidence and oblique incidence, respectively. The detection angle changes from 0° to ±40°.

To demonstrate angle relevant coloration of the fabricated wing scales, reflectance spectra were scanned from various viewing angles by an angle-resolved microspectroscope (ARM-51M; Ideaoptics Instruments Co. Ltd., China). As schematically shown in Fig. 7, the incident angle of light was fixed at either 0° or 30°, and the reflected spectra were collected by a 100× objective lens (NA = 0.8) at the angle between −40° and 40° to the normal.

**Figure 8:**
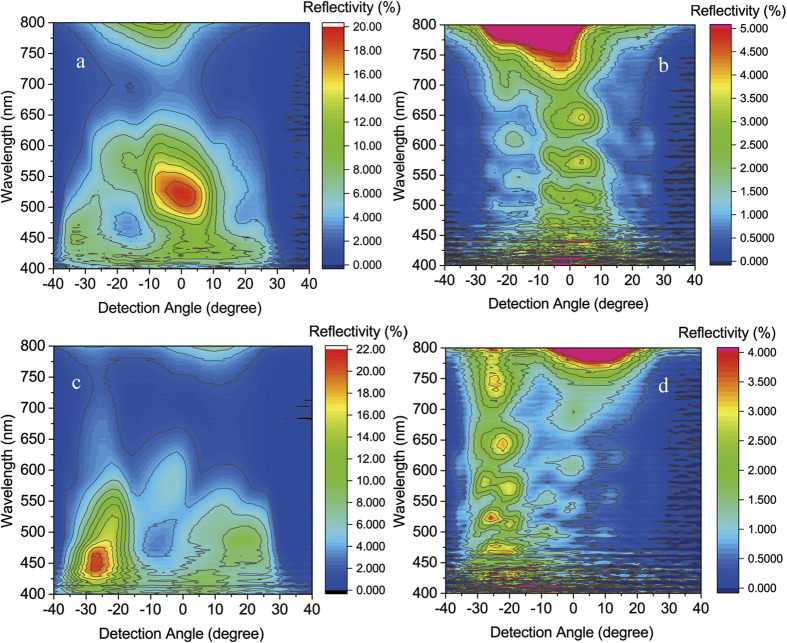
The measured angle-resolved reflectance spectra from the fabricated green color scales with totally 15 layers (Green_3) under normal incidence (a) and oblique incidence (c), respectively. The detection angle changes from 0° to ±40° progressively. For comparison, the same measurements were repeated on PMMA grating (**b,d**). Detailed descriptions are given in the text.

Figure 8a presents the 2-D map of spectra taken at the angles from −40° to 40° with normal incidence of illumination on Green_3. The reflection peaks are mainly around 523 nm (green color). The intensity of reflections decreases to nearly half for the viewing angle increases from the normal to ±16°. This indicates that the fabricated scale is able to hold green color in the viewing angle range of 30°. When the viewing angle is further increased, the overall spectra undergo a slight redshift by 48 nm from 523 to 571 nm, which is attributed to the contribution by the periodical ridge grating, as will be discussed later. Figure 8b shows the 2-D map of spectra from a PMMA grating with the same dimensions in pitch, height and duty ratio as sample Green_3 for comparison. A completely different nature of coloration is observed. In the visible region, there are two weak peaks around 575 and 650 nm, respectively, and the majority is located above 750 nm. With the detection angle moves away from the normal, the peaks shift toward long wavelength, proving that the ridge grating should be responsible for the redshifts observed in the fabricated Green_3.

Similar coloring behavior was also repeated for oblique incidence (Fig. 7) of lights in green color, as shown in Fig. 8(c,d). But the reflectivity is maintained in a smaller angle range (about 15°) than in the normal incident of light. A blue shift in zero order reflection is seen when the incident angle is increased, following the multilayer reflection characteristics^14^. The reflectivity map by the PMMA grating is also shown in Fig. 8d. With oblique illumination, the reflectivity once again shows typical interference pattern in a number of wavelengths corresponding to a series of harmonic resonances. Its component in green region is very limited.

## Discussions

Owing to the limitation of fabrication technique, there are still a number of differences in the structures between the fabricated and real wing scales, leading to deviations in coloration. First, the ridge pillars are formed by different refractive index materials (PMMA and LOR), which causes the reduction of reflectance by 10–20% with some dips on the peaks arising from resonant transmission modes in the PMMA/LOR pillar; Second, the artificial scales have strong regularity in the structure including periodical ridge grating with the same height and flat top. As the result, red component by the ridge grating is added to the total color by 10%, leading to slight redshift with oblique viewing angels. Furthermore, the angle independence of blue color is narrowed down to roughly ±16°. Third, the material in the artificial scale is polymer based and totally colorless. In this issue, no observable effect is detected, indicating that the blue color in this work is entirely caused by the lamellae structure free-standing on the scale without the need of pigment. Finally, the total periods are 7 with 15 layers, which is much less than the real ones. This is believed to be the main reason for low reflectivity in the artificial wing scale. Nevertheless, the observed coloring property by the mimicked scales provide us with invaluable experimental evidences in the interpretation of the blue iridescence seen from real butterfly wings. More importantly, it points out the direction in technical development to improve the process and the design by creating necessary irregularity in real scales. For example, the surface of the substrate can be pre-treated to create random mesas with the heights in the order of 50 nm. Fluorine based dry etch in plasma can naturally file the top corners of each tree in the ridge grating to form Christmas-tree like shape. Therefore, we believe, with further improvements on the nanolithography process innovated in this work, the artificial butterfly wings should mimic real ones closely, leading to promising applications in the future.

## Methods

### The finite difference time domain simulation

In the simulation of coloration by the constructed wing-scale, we used the FDTD Solutions software supported by Lumerical Solutions Corporation. A collimated source emits a spectrally broad (400–700 nm) plane wave with both TE and TM polarization components. In horizontal direction the model is extended to infinity with periodic boundary conditions (BC) and it in vertical direction are absorbing (perfectly matched layer, PML). Both SiO_2_ and Si are used as substrates to compare the reflectivity, and their refractive indices are taken from Palik^42^.

### The process flow for the fabrication of aligned lamella layers

**Figure 9:**
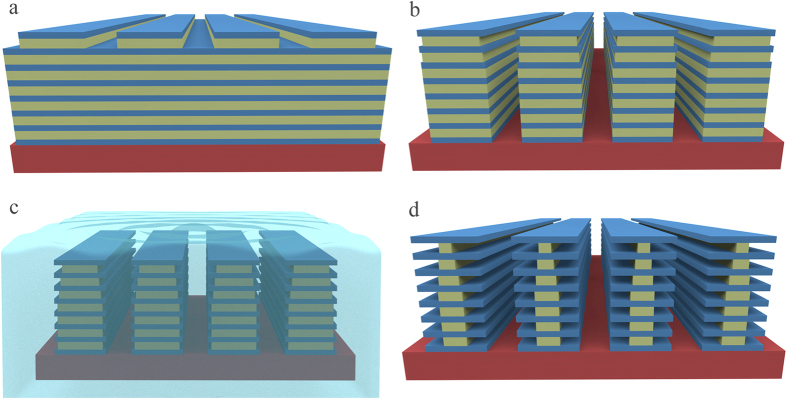
The process flow of the nanofabrication of artificial scales with lamellae layers free-standing on wing scale by electron beam lithography combined with alternate development/dissolution of PMMA/LOR multilayers. (**a**) E-beam exposure followed by a development on PMMA by the developer of MIBK:IPA and a dissolution on LOR by an alkali solution. (**b**) A ridge grating is formed after EBL before the final dissolution in alkali solution. (**c**) A final dissolution process creates PMMA/Air branches as lamellae multilayers. (**d**) The fabricated wing scale with aligned lamellae layers.

Figure 9 schematically shows the major steps of the process. Multilayers of PMMA/LOR are alternately spin-coated on silicon and quartz substrates, respectively, with designed thicknesses as shown in Fig. 9a. The resists were baked in oven after each coating in turn. Direct e-beam write was carried out by an e-beam writer (JEOL 6300 FS; JEOL Ltd., Japan) at 100 kV with a beam of 7-nm spot-size and 500 pA current under the tension of 100 kV. The written patterns were gratings with the pitch of 1.3 μm (for blue color) and 1.4 μm (for green) with a dosage of 800 μC/cm^2^. The overall area of each pattern is 1000 × 200 μm^2^. After e-beam exposure, a series of alternating development (for PMMA) by MIBK/IPA and dissolution (for LOR) by alkali CD26 were carried out, opening up deep trenches as shown in Fig. 9b. A concentrated CD26 solution (Shipley Corp.) was finally applied to selectively dissolve the LOR layers for creating undercuts with the assistance of ultrasonic agitation, as shown in Fig. 9c. The undercut width was controlled by dissolution time. Inspections of the layer structures were done by a Zeiss SIGMA HD scanning electron microscope. Optical images were acquired by a Zeiss Axio Scope A1 optical microscope.

## Additional Information

**How to cite this article**: Zhang, S. and Chen, Y. Nanofabrication and coloration study of artificial *Morpho* butterfly wings with aligned lamellae layers. *Sci. Rep*. **5**, 16637; doi: 10.1038/srep16637 (2015).

## Figures and Tables

**Table 1:**
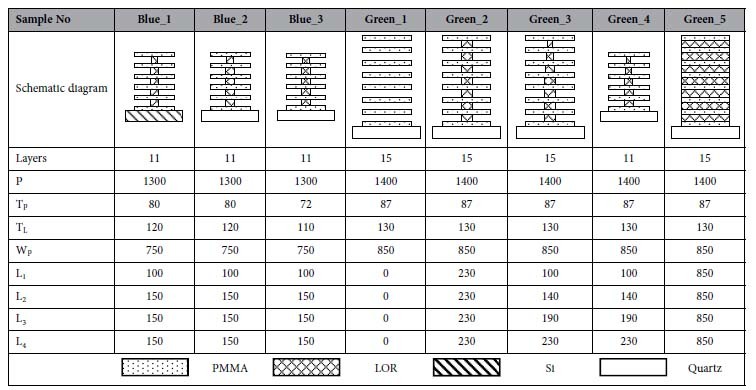
The sample names and the corresponding schematic diagrams of the designed and fabricated lamellae layers on artificial wing scales are all listed in the table.

The dimensions of the ridge grating period (P), layer thickness (T), branch widths (W) and pillar width (L) are all in nanometer.
